# Anthropomorphic Phantoms for Confirmation of Linear Accelerator-Based Small Animal Irradiation

**DOI:** 10.7759/cureus.254

**Published:** 2015-03-05

**Authors:** Julian R Perks, Steven Lucero, Arta M Monjazeb, Jian Jian Li

**Affiliations:** 1 Radiation Oncology, UC Davis Medical Center; 2 Biomedical Engineering, UC Davis

**Keywords:** 3d printing, linear accelerator small field radiation

## Abstract

Three dimensional (3D) scanning and printing technology is utilized to create phantom models of mice in order to assess the accuracy of ionizing radiation dosing from a clinical, human-based linear accelerator. Phantoms are designed to simulate a range of research questions, including irradiation of lung tumors and primary subcutaneous or orthotopic tumors for immunotherapy experimentation. The phantoms are used to measure the accuracy of dose delivery and then refine it to within 1% of the prescribed dose.

## Introduction

The small animal model is one of the cornerstones of biological research, often being used as a precursor to human Phase 0 and Phase I trials [1]. In the field of radiation oncology, the research design may well involve irradiation of the small animal with either lethal or sub-lethal doses. Additionally, the experimental design may require irradiation of a particular body part or system rather than the whole animal. In the case of the mouse model, the use of fields smaller than the whole body requires specialized dosimetric techniques and innovative design to ensure the radiation dose is delivered with sufficient accuracy to provide reliable and trustworthy experimentation.

The purpose of this paper is to document the support of radiobiological small animal research by a modern radiation oncology facility. There are many options for small animal irradiation, including dedicated X-ray sets for whole body irradiation [2-3] and even devices for sub-millimeter fields [4]. An issue with dedicated devices, however, is the associated costs of capital investment and upkeep. By utilizing existing technology from the clinic, the radiobiological researcher can take advantage of accurately commissioned and maintained equipment. By tailoring the irradiation to the experiment in terms of field size and energy, it should be possible to fulfill the requirements of the research, the exception being irradiations on the order of millimeters in size. The use of the sub-millimeter, precision-type animal irradiators [4-6] is beyond the scope of this paper. Precision, millimeter field size, imaging, and treatment of mice is certainly a significant step in radiobiological research; however, as the experiments documented below show, there are major areas of research which require field sizes larger than those of precision devices.

The modern radiation oncology clinic is equipped with high-energy linear accelerators, CT scanners, and, in rare circumstances, other imaging devices, such as MR or PET systems. Typically, the devices are optimized for human use, although modern radiotherapy practice relies heavily on small field dosimetry for techniques, such as intensity-modulated radiation therapy and stereotactic body radiation therapy, the understanding being that small fields are 3 cm x 3 cm or smaller. The availability of kilovoltage (kV) machines for small animal irradiation may be limited as kV sets are progressively being removed from clinical radiation oncology practice.

When linear accelerator-generated small fields are used for partial body irradiation in murine models, special considerations need to be applied, as the standard beam data used for human treatments cannot be accurately extended to fields approximately 2 cm to 3 cm [7]. Particular care has to be taken to confirm the output of the field as this ensures accurate dose. Changes in the percentage depth dose have to be considered so that the dose is delivered to the correct location. For each of the experiments documented in this work, a specific dosimetric check has been performed, with either additional calculations or in vivo measurements to confirm the accuracy of dose delivery.

In order to give the most accurate dose delivery to the small animal, the principles of human irradiation have been applied in the form of a dose prescription. In humans, dose is prescribed by stating the amount of radiation (gray), the number of times the dose is applied (fractionation), and the anatomical site and dose limits to surrounding critical structures. The dosimetric task of the treatment team is then to interpret the prescription to give the highest ratio in terms of accuracy of dose delivery and minimal dose outside the target area. A number of parameters related to the radiation beam can be altered within the limits of the radiation producing devices available. These beam parameters include energy, field size, and field shape.

In the radiation oncology clinic, the delivery will be by a linear accelerator that can produce high energy X-rays and accelerated electrons. For small animal irradiation, only the lowest energies will be useful, namely 6MV X-rays and 6MeV or 9MeV electrons. 

## Materials and methods

Ethics Statement:

All mouse studies described in this paper have been approved by the University of California, Davis Institutional Animal Care and Use Committees (IACUC). The University of California, Davis Institutional Animal Care and Use Committee (IACUC) issued approval #17090. Humane endpoints as specified by IACUC guidelines were utilized, including but not limited to: tumor burden greater than or equal to 10% of the animal's normal body weight, tumors exceeding 2 cm in size, a 20% decrease in body weight, inability to reach food or water, or a body condition score less than 2 on a 5 point scale. Mice were monitored daily (or more often if needed) during the study period.

All measurements in this work were made on an Elekta linear accelerator, configured with an Agility head (Elekta Ltd., Crawley, West Sussex, England).

**Figure 1:**
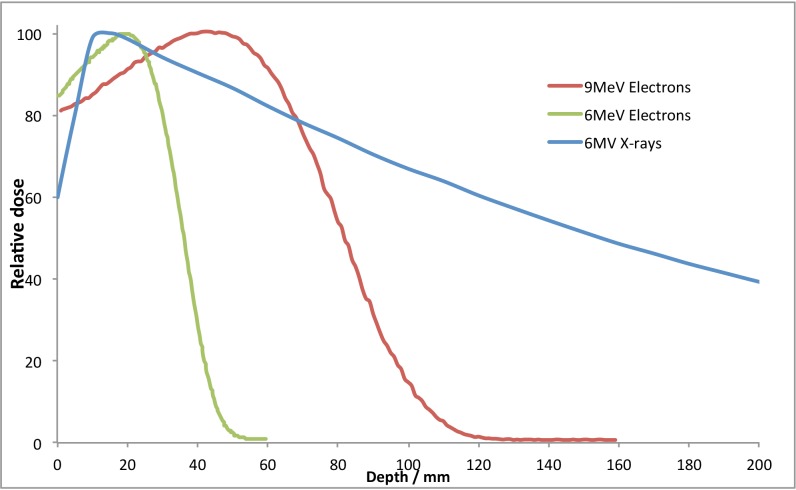
Percentage depth dose plots for the 6MV photons, 6MeV and 9MeV electrons used in this study

The percentage depth dose characteristics of these beams are given in Figure 1.

From Figure 1, it can be seen that neither the X-ray nor the electron beam will give a full dose to the surface of the animal; some build up or bolus material will be required. Bolus material (Superflab, Mick Radio-Nuclear Instruments, Inc. Mount Vernon, NY, USA) is used in either 0.5 cm or 1 cm thickness to bring the point of maximum dose deposition closer to the patient surface by absorbing radiation before it reaches the skin and initiates scattered electrons. All irradiations were carried out with a 5 cm solid water (Gammex, Middleton, WI, USA) block under the real or phantom mouse to provide a consistent amount of backscatter.

For this article, four scenarios are presented to demonstrate the breadth of available prescriptions and their respective dosing strategies. All of the scenarios were delivered to mice and no other animal was considered for this paper. However, as mice are generally accepted as the archetypal small animal model, the methods presented should be widely applicable. Additionally, for larger animals, the dosimetric principles of the modern linear accelerator should be easier to apply – the larger the animal, the closer the dosimetry will be to human principles.

The scenarios presented for analysis are:

Whole Body Dosing

This is the most widely established protocol and is used for a variety of research questions [8-10]. One such experiment is to investigate chronic and acute graft versus host disease in mice undergoing bone marrow transplant. The mice are irradiated in a plastic cage, usually with more than one animal at a time, using a wide field of 20 cm x 20 cm with 1 cm of bolus material over box.

Lung Irradiations

The lung and gut irradiations were used for organ-specific toxicity research or with a similar intent of eliciting a graft vs. host response, but from a specific anatomical location rather than total body [11]. Lung irradiations were also used to investigate treatment of metastatic or primary lung tumors. For the lung irradiations, a 9MeV electron field was used with 1 cm bolus material. Electrons were initially chosen to compensate for the low-density tissue in mouse lung, and a single anesthetized animal was irradiated at a time. A small, cadmium-free, low melting point alloy (Radiation Products Design Inc, Albertville, MN, USA) 3 cm x 3 cm cutout block was used to shape the electron field, with the output factor of the cutout measured as 0.95. The PDD curve for the 3 cm x 3 cm cutout was confirmed with end-on irradiated film.

Gut Irradiations

This irradiates a thicker portion of the animal with no low-density tissue so 6MV X-rays were selected, and as with the whole body irradiations, the 1 cm bolus material was used. The photon beam characteristics, tabulated at the time of linear accelerator commissioning for human use, allowed a long, narrow, asymmetric field to be used; a 20 cm x 5 cm field was used so that multiple animals could be irradiated at once. The field was designed so that the divergent edge of the field fell across the animal’s tail, with a perpendicular edge shielding the anterior aspect of the mouse.

Conformal, Small Fields

Focal irradiation was used to treat tumor-bearing mice in which we desired to treat the tumor, but not the whole animal. Such experiments are particularly important when the role of the immune response to radiotherapy is being considered, as TBI is systemically immune suppressive. The animal was prepared with one or two subcutaneous or orthotopic lesions. The dose prescription called for one of the lesions to be irradiated whilst sparing as much of the remaining animal. Six MeV electrons are chosen with 1 cm build-up to give a full dose to the tumor with the fastest depth dose fall-off available to minimize the dose to the remainder of the animal.

### Confirmation measurements

The individual dosimetric plans for the four scenarios above were confirmed by measurements. In vivo measurements in sacrificed animals were considered, but a cleaner and more reproducible solution was found. To confirm the doses, a set of “phantom mice” was designed and constructed using 3D printing technology. A toy mouse was laser scanned using a Nextengine 2020i scanner (Nextengine, Santa Monica, CA, USA) to yield a virtual model through Netfabb v.4.9 and v.5.0 (Netfabb, Lupberg, Germany) and Autodesk Inventor Professional 2014 (Autodesk, San Francisco, CA, USA) software. The mouse model was then modified to add holes to hold ionization chambers or MOSFET (metal oxide silicate field effect transistor) detectors and to simulate lungs. Seven different phantom mice were printed for this study; this covered the range of experiments for whole body, lung, gut, and flank tumor irradiations. The phantom mice were 3D printed on Objet Eden and Connex printers (Stratasys, Eden Prairie, MN, USA) with VeroClear photo-polymer (Stratasys, Eden Prairie, MN, USA). The 3D printing process jets the polymer in 16 µm layers, with each layer hardened by ultraviolet light. The manufacturer stated accuracy of the print is 20 – 85 µm for features below 50 mm, and 200 µm for the entire printed object.

The ionization chamber chosen to measure the dose for this work was the A1SL (Standard Imaging, Middleton, WI, USA), a standard radiation therapy tool for small field, high-energy radiation measurements; it has a 0.053 cm^3^ active volume. The charge collected by the ionization chamber was recorded by a Keithley 614 digital electrometer (Keithley Instruments, Inc., Cleveland, Ohio, USA). Both the chamber and the electrometer were calibrated by the University of Wisconsin’s Accredited Dosimetry Calibration Laboratory. The radiation dose was measured according to the American Association of Physicists in Medicine protocol TG51 [12].

The MOSFET detectors for the flank tumor measurement (Best Medical, Ottawa, Canada) were used with the standard resolution bias; these detectors have a 0.2 mm x 0.2 mm active volume. The MOSFET was not used for all measurements due to its lower reproducibility of measurements (2%).

The dose measurements were performed as follows:

CT Scans

To confirm the mouse phantom is representative of the animal, measurements of weight and density were made. To check the equivalency of the mouse phantom to the live creature, the phantom was CT scanned and analyzed using standard radiation oncology treatment planning software (Pinnacle v. 9.2, Philips Healthcare, Andover, MA, USA). Four anesthetized mice were also scanned with the same setup and again analyzed with the Pinnacle software. The density of various components were measured, and the weights of the real mice were also compared to the phantom mice.  

Whole Body Dosing

**Figure 2:**
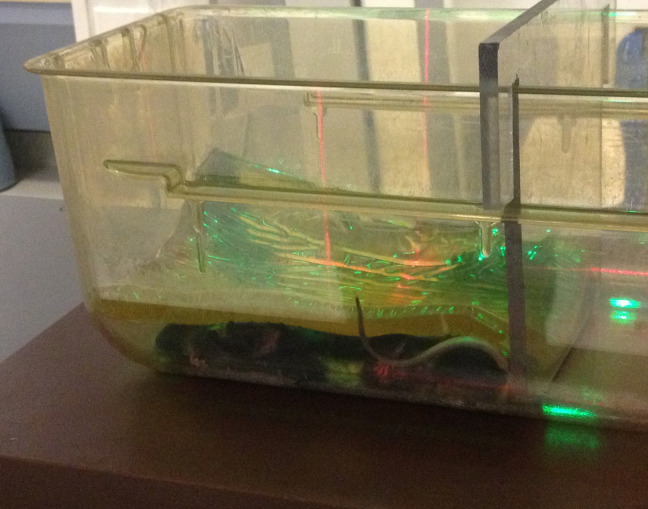
Mice in position for whole body irradiation, under bolus at linac room isocenter (as marked by room lasers)

A 3D-printed mouse with the chamber hole in the center of the body was irradiated in multiple positions under the beam with bolus directly on top. Additionally, the dose inside the phantom mouse was measured when research mice were irradiated in their cage with the bolus material on top, the standard whole body setup. Figure 2 shows the setup for whole body measurements in a standard cage.

**Figure 3:**
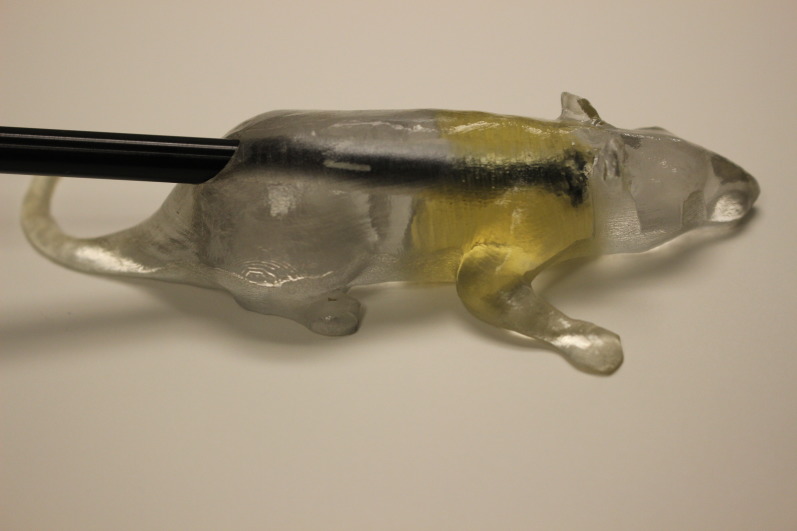
3D printed mouse phantom with A1SL ion chamber, used to confirm dose in whole body irradiation

The 3D-printed mouse was remodeled to include representations of lungs with the chamber hole directly between both lungs. The lung was modeled by a grid pattern of printing material with air gaps, approximating one-third density tissue. Figure 3 shows the setup for lung irradiations.

A shorter chamber hole was designed for the 3D-printed mouse that measured the gut irradiations, midway between anterior and posterior surfaces. Lungs were not simulated in this model. There are three sets of readings: central axis, left-hand, and right-hand sides of the field.

**Figure 4:**
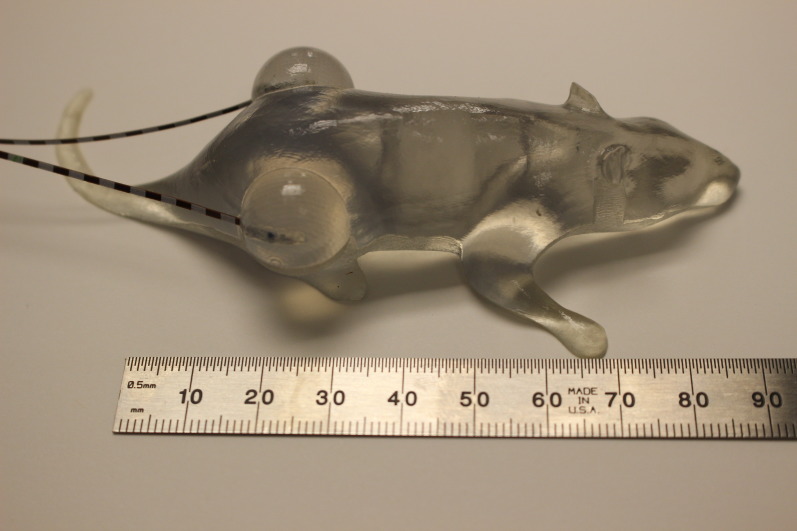
3D printed mouse phantom with MOSFET detectors for confirmation of focal electron radiation

Finally, for the small field, conformal surface dose measurements of the same mouse as whole body were used, but with a plug to fill the chamber hole. MOSFET detectors were placed on each side. By then irradiating one side of the model with bolus, the dose to both the planned target and the non-irradiated target were simulated. The MOSFET detectors were calibrated in 6MV X-rays and are known to be energy independent. Figure 4 shows the setup for subcutaneous tumor irradiations.

The measurements in the phantoms were used to validate and confirm the doses prescribed to the experimental white mice. The phantom measurements also allowed refinements of the monitor unit settings when the dose to the mouse, calculated from the standard human beam data, differed by more than 1% from the expected amount. 

## Results

The weights and physical density properties of the real and phantom mice are documented in Tables 1-2. The aim of printing a mouse with "lungs" was to have a space with only two-thirds print material by volume, and this was confirmed with the CT scan.

**Table 1:** Density measurements from real and phantom mice The electron density measurements of the real and 3D printed phantom mice, showing the tissue equivalence necessary to derive the guidelines for accurate irradiations.

Scanned area	Density g/cm^3^
Solid water	1.06
Bolus material (superflab)	1.00
Phantom mouse material	1.15
Phantom mouse (lung)	1.06
Mouse gut	1.12
Mouse lung	0.66
Mouse bone	1.21

**Table 2:** Weights of real and phantom mice assessed by CT scanning The weight (in grams) of the real and 3D printed phantom mice used in the research and confirmation measurements, respectively.

Description	Weight / g
Live, Balb/c mice (average of four animals)	22.2 (average) 19.8 – 24.5 (range)
Whole body phantom	31.2
Lung phantom	31.1
CT phantom	32.9
Subcutaneous phantom	35.4

The phantom mice were somewhat heavier than the average white mouse, but the density of the phantom material compared to density range across the real mice showed that the printed material would yield sufficiently accurate measurements of ionizing radiation.

The measured doses, compared to the prescribed doses, in conjunction with the type of radiation used are given in Table 3.

**Table 3:** Prescribed and measured doses for the mouse irradiations A comparison of the expected and measured dose (in Gy) for each mouse phantom

Setup of Phantom Irradiation	Energy	Prescribed dose / monitor units	Measured dose (Gy)	Comments
Whole body, 1 cm bolus directly on mouse	6MV X-rays	2Gy / 191MU	2.001	
Whole body in cage, 1 cm bolus material draped over cage	6MV X-rays	2Gy / 191MU	1.968	1.7% lower due to loss indirect bolus
Mouse gut, 1 cm bolus, half blocked field	6MV X-rays	2Gy / 203MU	2.003	Average measured dose from three positions
Mouse lung, solid mouse, 1 cm bolus, 3x3 cm field size	9MeV electrons	2Gy / 227MU	1.958	2.1% lower than prescribed
Mouse lung, solid mouse, 1 cm bolus, 3x3 cm field size	9MeV electrons	2Gy / 223MU	2.001	Monitor units adjusted
Mouse gut when lungs are irradiated, 1 cm bolus, 3x3 cm field size	9MeV electrons	2Gy / 223MU	2.022	1.1% increase in measured dose due to lower attenuation in lung area
Mouse lung, mouse with lungs, 1 cm bolus, 3x3 cm field size	9MeV electrons	2Gy / 223MU	0.178	Equivalent of the 9% isodose line
Tumor on mouse flank, 1 cm circular field,	6MeV electrons	1Gy / 147MU	0.957	MOS / FET detector
Tumor on mouse flank, 1 cm circular field,	6MeV electrons	1Gy / 155MU	1.015	Adjusted monitor units
Contralateral tumor mouse flank	6MeV electrons	1Gy / 155MU	0.04	Equivalent of the 4% isodose line

With a prescription of 2 Gy, the dose measured for the whole body dosing was 2.001 Gy when the bolus material was placed directly over the phantom mouse, and 1.968 Gy when the phantom mouse was placed in a standard plastic cage with the bolus material draped over. In order to deliver the required dose as prescribed, the monitor unit setting on the linear accelerator was increased from 191 MU to 194 MU. This accounts for the loss of scattered radiation in the air gap between the bolus and the phantom.

Using the mouse phantom with low-density material representing lungs, a prescribed dose of 2 Gy with 9MeV electrons through a 3 cm x 3 cm field was measured at 1.958 Gy, requiring an adjustment of monitor units from 227 MU to 233 MU. With the lung field set up, the gut of the phantom mouse was placed under the beam. This showed that the dose outside the lung field to the remainder of the animal is very low at 0.178 Gy. 

The gut phantom measurement required no adjustment of monitor units as 2.004 Gy was measured with the 6MV X-ray offset field, with 1 cm bolus laid directly over the phantom.

The flank tumor phantom was irradiated with 6MeV electrons, through a 1 cm cut out with 0.5 cm bolus. The dose, measured by MOSFET detectors, was 0.957 Gy for a prescribed dose of 1 Gy.  An adjustment of monitor units, from 147 to 155 MU, gave a measured dose of 1.015 Gy. With the phantom mouse set for flank irradiation of 1 Gy, the contralateral flank was measured at 0.04 Gy, demonstrating the extremely rapid fall off of dose from the low energy electrons used. 

The summary of our measurements and a guide for all the mouse irradiations we performed is given in Table 4.

**Table 4:** Guidelines for mice irradiations The appropriate linac settings (in monitor units) for each experiment.

Scenario	Prescription and setup	MU / Gy
Whole body	Mice in cage, cage on 5 cm thick block of solid water, couch raised to set laser isocenter at mid plane of mouse, 1 cm bolus over cage, 6MV X-rays, 20x20 cm field (scatter factor 1.05), prescription dmax at mouse midplane.	97
Lung	Anaesthetized individual mouse on 5 cm solid water block, couch raised to set laser isocenter at mid plane of mouse, 3x3 cm electron cutout, 1 cm bolus directly over mouse, 9MeV electrons, prescription dmax at lung central plane.	116.5
Gut	Anaesthetized line of mice on 5 cm solid water block, couch raised to set laser isocenter at mid plane of mouse, 1 cm bolus material directly over mouse, 6MV X-rays, 5x20 cm field (scatter factor 0.984), prescription dmax at gut midplane.	100
Subcutaneous/Orthotopic tumor	Anaesthetized individual mouse on 5 cm solid water block, tumor requiring radiation at laser isocenter, linac gantry angled to provide optimal coverage, 0.5 cm bolus over tumor, 6MeV electrons, 2 cm circular cutout, prescription at tumor center.	153

## Discussion

This work has demonstrated a strong role for the standard, clinical linear accelerator in small animal research, facilitating standard whole body dosing as well as small field, conformal treatments down to 1 cm field size. The accuracy of measured dose, when the prescribed dose had been translated to a monitor unit setting via measured beam data, was always within 5%. Our action level for adjusting monitor units, based on the phantom measurement, was a difference of 2% or higher. The electron irradiations of the phantom lungs and the flank tumors needed adjustment; the anthropomorphic phantoms allowed refinement of the initial output factor measurements for these fields, which was made in a large block of solid water.

The linear accelerator used in this work is a standard, clinical machine for human radiation oncology, used on a daily basis. As it is in a university hospital setting, it is used for research purposes after clinical hours, although the properties of the radiation are not altered. For small animal research, individual fields are designed and measured according to the researcher’s prescription [13]. The beam energy of the accelerator is typically an order of magnitude (MeV c.f. 100keV) higher than dedicated small animal irradiators; however, as this work demonstrates, accurate doses can be achieved with carefully designed beam modifiers (field size and bolus). Additionally, very low doses can be conformed to the remainder of the animal when necessary, as demonstrated by the gut measurement when the lungs were irradiated with electrons. One distinct advantage for the researcher in utilizing a clinical machine for irradiations is that he/she can rely on a rigorous maintenance and quality assurance schedule, ensuring irradiations are consistently accurate and the machine will be available with a better than 98% uptime.

The advance in technology, which made this work possible, was the 3D scanning and printing. This exciting and emerging field is rapidly being applied to medicine with a wide range of applications [14-15]. The current limits are manufacturing costs and print size. At the time of this writing, a 3D printer, with the required accuracy for medical applications, costs approximately $220k. The 3D-printed models require before-build material and support material; for the mice in this study, 41 gm of build material at $5 per 10 gm, and 17 gm of support material at $3 per 10 gm were used. The printer used to make the mice has a maximum print size of 255 mm x 252 mm x 200 mm.

## Conclusions

In conclusion, 3D scanning and printing technology facilitated a set of phantoms, which showed that conformal, small-field dosimetry for a mouse (and therefore larger animals) is achievable with 1% accuracy from a clinical linear accelerator. This demonstrates that many of the research scenarios, in which small animal irradiation is required, can be reasonably achieved with the use of a clinical-grade linear accelerator. An extension of this work would be to check the accuracy of the latest micro-beam small animal irradiators with anthropomorphic phantoms.
